# Blue laser for the exclusive endoscopic transcanal approach to middle ear paraganglioma

**DOI:** 10.1007/s00405-024-08470-x

**Published:** 2024-02-09

**Authors:** Mireia Quer-Castells, Marta Sandoval, Francisco Larrosa

**Affiliations:** 1 Department of Otorhinolaryngology-Head and Neck Surgery, Hospital Clinic de Barcelona, C Villarroel 170, 08036 Barcelona, Spain; 2 University of Barcelona Medical School, C Villarroel 170, 08036 Barcelona, Spain

**Keywords:** Glomus tympanicum, Paraganglioma, Middle ear, Transcanal, Endoscopic ear surgery, Blue laser

## Abstract

**Background:**

The management of glomus tympanicum tumours can be challenging. Blue laser coagulation may improve bleeding control thus facilitating an endoscopic transcanal excision. The objective of this presentation is to illustrate the authors’ experience using this novel tool.

**Methods:**

Case report of a patient that underwent exclusive endoscopic transcanal blue laser surgery of a class A2 glomus tympanicum tumour in a tertiary referral center.

**Conclusion:**

The present study provides evidence of the safety and efficacy of endoscopic blue laser surgery, for the minimally invasive treatment of early-stage glomus tympanicum tumours.

## Introduction

The transcanal endoscopic approach to middle ear paragangliomas is gaining popularity. Minimally invasive surgery is allowed by improved visualization, thus reducing postoperative morbidity [1, 2]. However, the one-handed, management of these bleeding lesions can be challenging. In addition, experience in this field is limited since these tumours are uncommon [3, 4].

Blue laser is a 445-nm semiconductor laser and is highly absorbed by the red and black spectrum (such as haemoglobin and melanin). It combines photoangiolytic and cutting properties in a single laser frequency [5]. It offers less bleeding and lower thermal damage compared to the infrared diode laser [6].

This is a case report to illustrate the current management of glomus tympanicum tumours using blue laser fibre through an exclusive transcanal endoscopic approach.

**Fig. 1:**
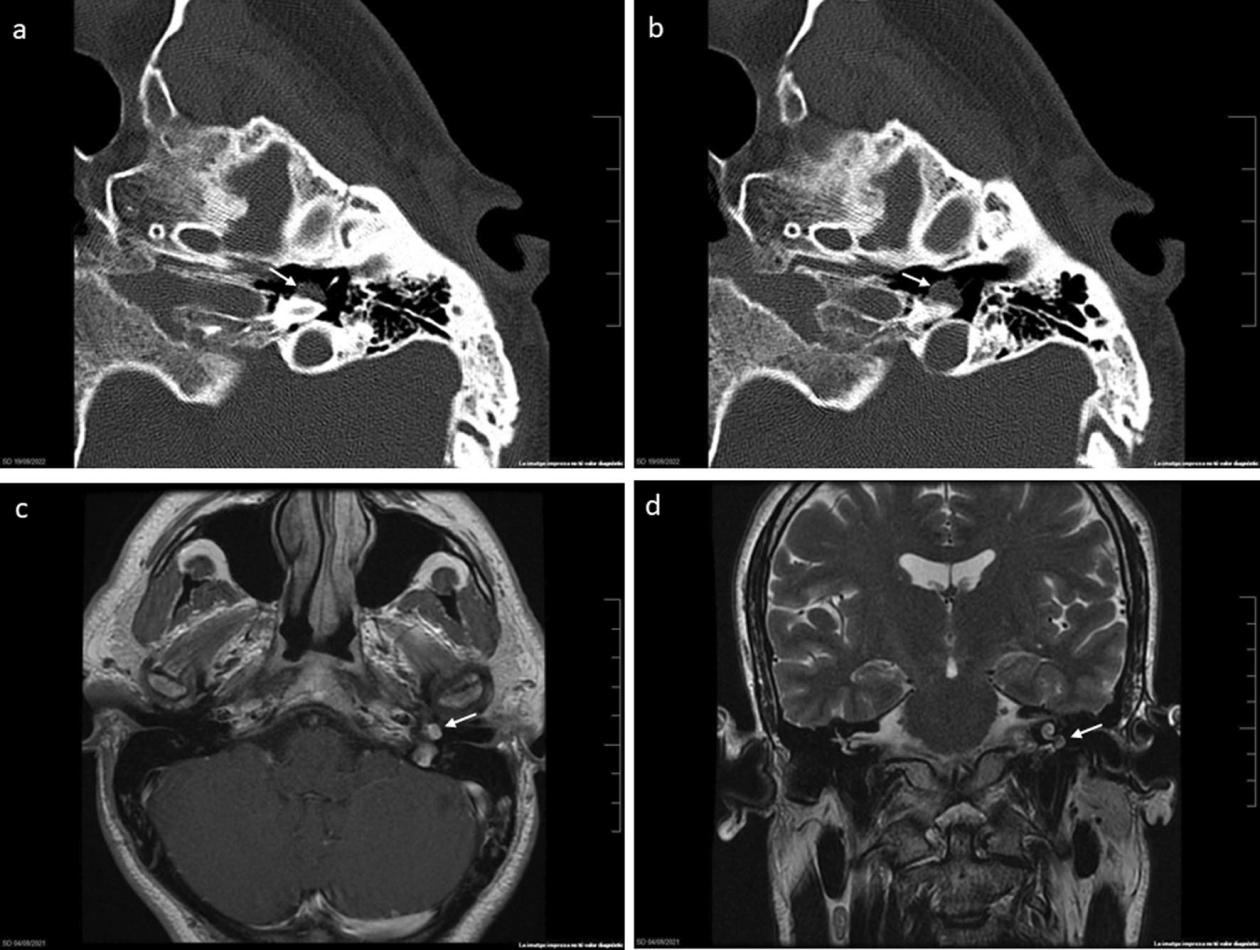
Axial CT scan images showing round lesion located on the promontory (**a**, **b**); Same lesion on axial MRI T1 with contrast (**c**); and coronal MRI T2 sequences

A 44-year-old man diagnosed with class A2 glomus tympanicum tumour, according to the modified Fisch-Mattox [7] classification, was referred to our university tertiary referral center for treatment. He complained of left ear pulsatile tinnitus and a redish retrotympanic tumour, margins not visible, was observed on otoscopy. Hearing was preserved. The CT scan revealed a round, soft tissue, mass on the promontory and the MRI showed an enhancing mass lesion extending anteriorly into the eustachian tube (Fig. 1a–d).

## Relevant surgical anatomy

Glomus tympanicum tumours are rare benign tumours commonly originating from the Jacobson’s or Arnold’s nerves, on the medial wall of the middle ear. These tumours commonly present with pulsatile tinnitus and conductive hearing loss. A redish retrotympanic mass is visualized. Differential diagnosis of retrotympanic lesions should include adenomas, schwannomas, congenital cholesteaomas and malignancies [1, 4]. Most descriptions place these tumours on the promontory. They can extend anteriorly, to the protympanum, involving the tensor tympani muscle, the Eustachian tube and the carotid artery. Inferiorly, the hypotympanum is frequently occupied and care should be taken to avoid damage to the jugular bulb. The epitympanic space, the ossicular chain and the retrotympanum can be involved in class B1 tumours. The entire facial tympanic tract should be controlled during the procedure [1, 2].

## Description of the technique

An exclusive transcanal endoscopic approach was performed under general anesthesia on September 2022. A 0°, 3 mm ø, 14 cm length endoscope (Karl Storz SE & Co. KG, Tuttlingen, Germany) was used. The procedure began with local infiltration of the ear canal (Ultracain^®^ Laboratorios Normon S.A., Madrid, Spain). Neurosurgical patties soaked in lidocaine (B. Braun 20 mg/ml, 10 ml) with epinephrine (B. Braun 1 mg/ml, 0.5 ml) were helpful in controlling intraoperative bleeding as well as lavages.

The surgical phases were: (1) Tumour exposure: The technique involved the elevation of an extense, anterosuperior based, tympanomeatal flap; (2) Tumour reduction: coagulation of the tumour surface by blue laser fibre (2 W, 0.08 s/pulse; 2.5W 0.12 s/pulse – Fibre 300 μ, Blue laser WOLF TruBlue, Neomed, UK) and otology handpiece; (3) Tumour dissection and excision from posterior to anterior, searching when possible the main vascular supply. The suction dissector (Karl Storz SE & Co. KG, Tuttlingen, Germany) was very useful at this stage of the surgery. Traction dissection helped in the final stage of the tumour extraction; (4) Haemostasis and vaporisation of tumour bed residues by a blue laser. In the last two phases of the procedure, three-handed work was performed (two surgeons). The procedure ended with the reinforcement of the tympanic membrane with temporalis muscle fascia placed in an underlay fashion and the replacement of the tympanomeatal flap. The packaging consisted of an absorbable gelatine sponge filling the external auditory canal and a silicone sheet (See video, Supplemental Digital Content 1, illustrating technique). Surgery time was 90 min.

**Fig. 2:**
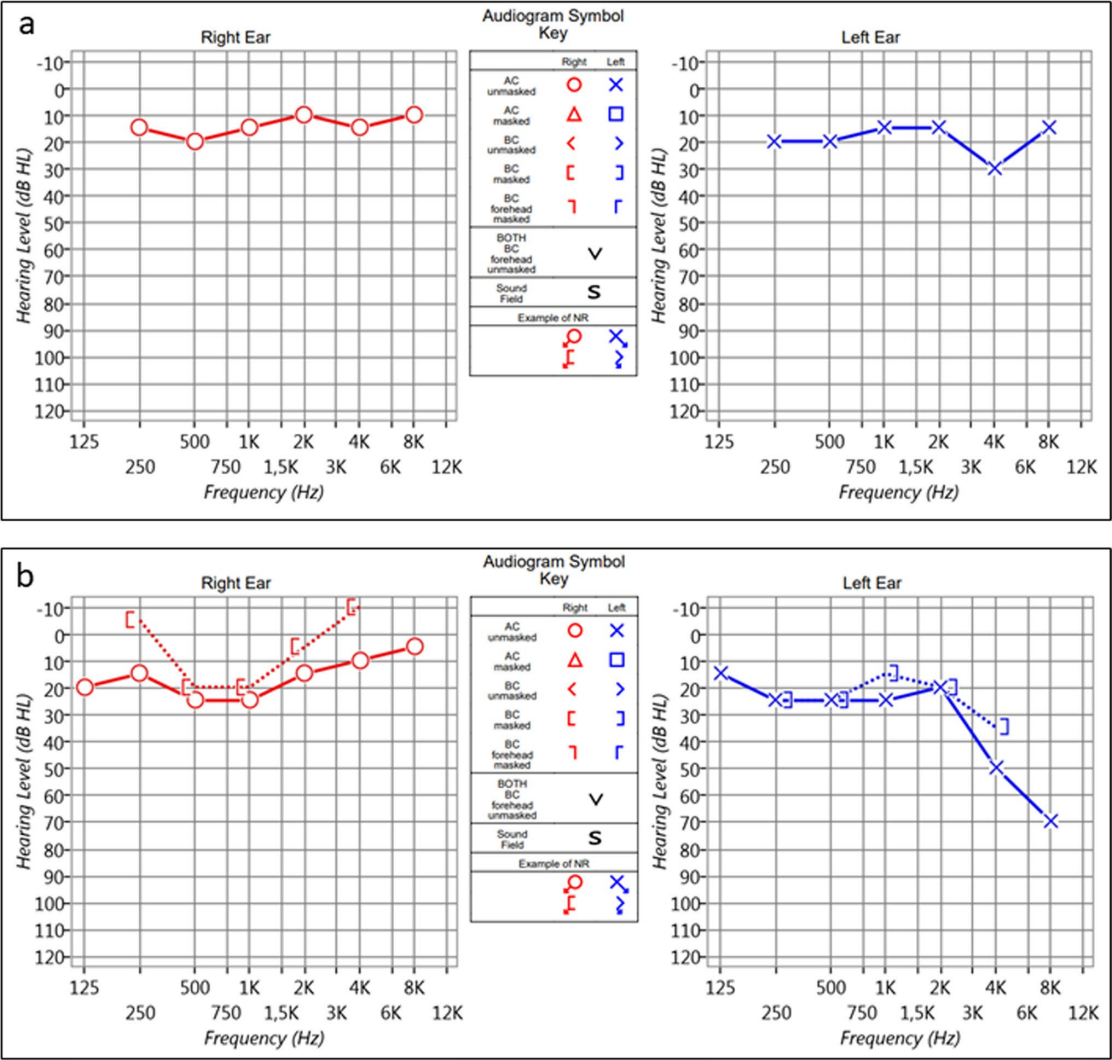
Preoperative (**a**) and 12-months postoperative (**b**) pure tone audiograms

The patient was discharged, without incident, on the same day. Histopathology confirmed the diagnosis. At one-year follow-up, the tinnitus disappeared although a mild sensorineural hearing loss was observed (Fig. 2a, b). Otherwise, no recurrence was observed.

## Indications

The exclusive transcanal endoscopic approach to glomus tympanicum can be a treatment option in tumours confined to the middle ear (classes A1–B1) [3, 4, 7].

## Limitations

Mastoid extension is a contraindication for exclusive endoscopic management. Thus, classes B2-3 are excluded from this treatment option.

## How to avoid complications

Preoperatively, dehiscences in the bony wall covering the internal carotid artery or a high, dehiscent jugular bulb may be detected on imaging studies (CT scan, MRI) thus preventing a possible catastrophic bleeding during surgery. Abnormalities in the facial nerve can be also assessed in this way. In view of those findings, facial nerve intraoperative monitoring may be considered. To avoid possible damage to neuroepithelial cells of the cochlea, causing neurosensorial hypoacusia, monopolar coagulation should not be used in the middle ear especially on the promontorium. Instead, bipolar or laser coagulation is recommended. Follow general safety rules for the use of lasers, comply with the manufacturer’s recommended settings for these neoplasms (power and pulse duration) and perform frequent irrigation to avoid excessive heat generation near the inner ear. In addition, care should be taken to set the light source intensity at 50% or less to avoid overheating of middle ear structures. To prevent a possible sensorineural hearing loss caused by ossicular manipulation during dissection of the tumor, some authors divide the incudostapedial joint to protect the stapes [8].

## Specific perioperative considerations

Preoperative imaging studies also provide valuable information on tumour extension, allowing the selection of the most suitable approach (transcanal vs. retroauricular). No specific postoperative recommendations need to be made other than the general ones after common ear surgery procedures. Patients should call the surgery centre in case of bleeding, facial palsy or severe vertigo.

## Specific information to give to the patient about surgery and potential risks

Should include conversion to a microscopic retroauricular approach under certain circumstances: limited space, excessive bone work or intraoperative complications such as uncontrolled bleeding or facial nerve damage. Additional potential risks should include ossicular chain trauma or disruption, inner ear damage, dysgeusia due to chorda tympani trauma and tympanic membrane perforation. The possibility of tumour recurrence should also be mentioned.

## Summary/key points

1. Glomus tympanicum is the most common benign tumour in the middle ear. Nevertheless, it is a rare tumour. The protympanic space is affected in most cases.
2. These tumours commonly present with pulsatile tinnitus and conductive hearing loss and a redish retrotympanic mass is visualized on otoscopy. Differential diagnosis of retrotympanic lesions should include adenomas, schwannomas, congenital cholesteaomas and malignancies.
3. The transcanal endoscopic approach offers a wider view of the middle ear, thus allowing minimally invasive surgery. However, the drawbacks of this approach are the narrow access and one-handed surgery, considering these are bleeding lesions.
4. The blue laser offers less bleeding and lower thermal damage on tissues. It allows to cut in non-contact mode with simultaneous coagulation.
5. The size and shape of the reusable handpieces turned out to be very convenient for the transcanal work.
6. Technique summarized in four steps: Tumour exposure (extense tympanomeatal flap), tumour reduction, tumour dissection and excision and haemostasis and vaporisation of tumour bed residues. Patties soaked in epinephrine, lavages and three-handed surgery (two surgeons) will help during surgery.
7. This approach is limited to class A1-B1 tumours, those confined to the middle ear. Class B2-B3 tumours are excluded from the exclusive transcanal endoscopic approach. A microscopic, retroauricular approach will be recommended in these cases.
8. To avoid complications a thorough preoperative assessment must include CT scan and MRI imaging both to assess tumour extension and possible anatomical abnormalities. Avoid using monopolar electrocautery in the middle ear. Consider dividing the incudostapedial joint to protect the stapes.
9. Information given to patients should include the possibility of conversion to a retroauricular approach and tumour recurrence.
10. The present study provides evidence of the efficacy and safety of the transcanal endoscopic blue laser approach for the current treatment of glomus tympanicum. Further studies are needed to confirm these findings.

## Data Availability

The data that support the finings of this study are available from the corresponding author upon reasonable request.
